# Primary Localized Vesical Amyloidosis Mimicking Bladder Carcinoma: A Case Report

**Published:** 2016-12-24

**Authors:** Purwa R. Patil, Bhushan M. Warpe

**Affiliations:** *Department of Pathology, Grant Government Medical College and Sir J.J. Group of Hospitals, Byculla, Mumbai - 400008, India.*

**Keywords:** Vesical Amyloidosis (VA), Urinary bladder, Congored

## Abstract

Amyloidosis of urinary bladder is a rare condition and may be primary or secondary in nature. A case of primary localized vesical amyloidosis (VA) in a 40-yr-old man is described which was confused with neoplasm by cystoscopic, urographic and other studies. Surgical specimens obtained by transurethral resection (TUR) were diagnostic and histologically revealed amyloid deposits in sub-epithelial stroma with chronic inflammatory and giant-cell reaction. Congo-red staining proved its amyloid nature. It was resistant to potassium permanganate (KMnO_4_) pretreatment, indicating it to be of the AL type.

## Introduction

Primary vesical amyloidosis is a rare clinic-pathological condition with approximately 210 documented cases in the world literature from 22 different countries (1). The largest number of such cases has been reported in the UK (60 cases) (1). In Indian literature, there is a mention of very few similar case reports (2-8). In its localized form, it is often indistinguishable clinically and cystoscopically from a bladder tumor before biopsy. Its most frequent presentation is painless hematuria (2-7). Exclusion of systemic amyloidosis is important both for management and prognosis, as localized primary vesical amyloidosis can be best treated by TUR, while diffuse form requires urinary diversion. Careful cystoscopic follow-up is required, as recurrence is known to occur (1). 

Here we report a case of primary localized vesical amyloidosis (VA) in a 40-yr-old man.

## Case report

A 40-year-old man presented with painless hematuria of two months duration. There was no history of long standing illness like significant joint pains or infectious disease. Patient had no history of dysuria, urosepsis, blood disorders, abdominal tenderness or tuberculosis. He did not have palpable abdominal mass or pelvic pain or inguinal lymphadenopathy. Informed consent was taken from the patient.

CBC findings showed: Hb-9 gm%, Total WBC count-6000/cu mm, Differential WBC count-N_68_L_28_E_2_M_2_, Platelet count-250000/ cu mm. Urine cytology revealed presence of RBCs and few degenerate ‘suspicious’ cells. Urine culture and sensitivity were sterile. Digital rectal examination revealed no prostatomegaly.

**Fig. 1:**
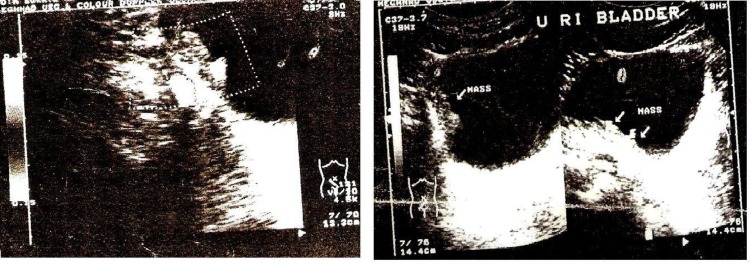
Pelvic USG showing mass along right lateral wall of bladder

USG abdomen-no abnormality detected. On pelvic USG, urinary bladder showed a cauliflower-like single mass measuring 3.1 x 2.9 x 1.9 cm in dimension arising from right lateral wall (Fig.1). CT-scan revealed a well-defined hyperdense enhancing single mass lesion, measuring 19 mm in thickness. There was no evidence of calcification, extra-vesical spread or lymphadenopathy. On imaging findings, possibility of carcinoma of bladder was suggested and cystoscopy and biopsy was advised for confirmation. At cystoscopy, there was single solid growth of three-centimeter diameter with surrounding erythematous lesion in right lateral wall of bladder. 

Serum proteins-8 gm/dl, serum total bilirubin-1.1 mg/dl, serum creatinine-0.9 mg/dl, serum ferritin-20 microgram/L, were all within normal limits. There was no evidence of Bence-Jones proteinuria by heat and acetic acid test of urine. Serum and urine electrophoresis did not reveal M-band and showed normal findings.

**Fig. 2:**
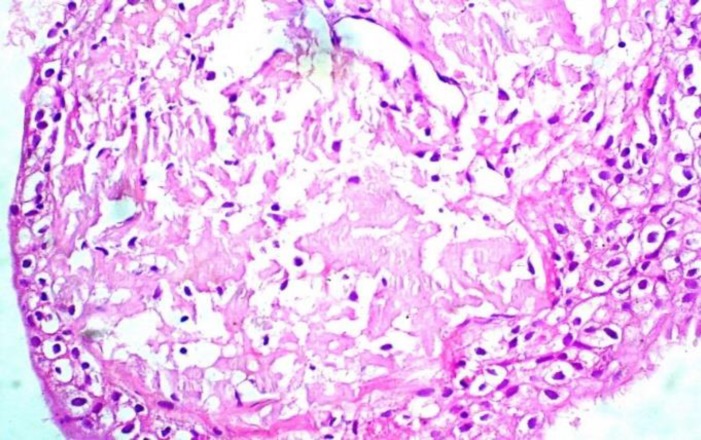
Microphotograph showing sub-epithelial interstitial deposits of amyloid in bladder wall (H & E, x 400

**Fig 3:**
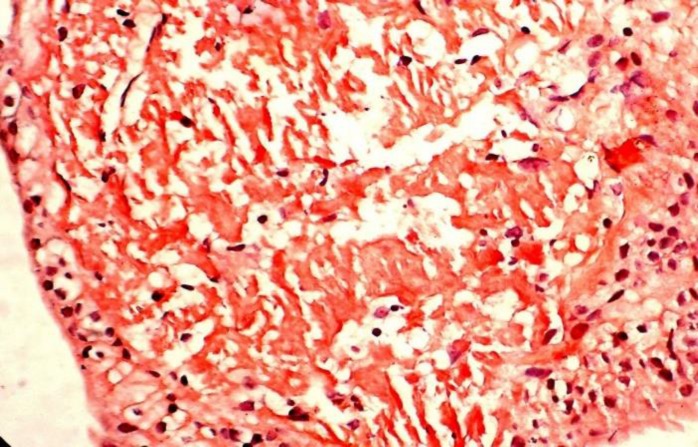
Microphotograph showing sub-epithelial interstitial deposits of amyloid in bladder wall stained with Congo red (Congo Red, x 400

The lesion was biopsied. Multiple tissue bits were received both at diagnostic and excisional cytoscopic biopsy. Formalin-fixed paraffin embedded tissue sections showed squamous metaplasia of lining epithelium and sub-epithelial interstitial deposits of homogenous eosinophilic material with foreign-body giant cell and chronic mononuclear reaction. Biopsy was superficial and included only mucosa, submucosa, which showed capillary-sized blood vessels with no obvious deposits in their wall (Fig.2). Congo-red staining proved its amyloid nature. It was resistant to KMnO_4_ pretreatment. This confirmed the primary nature of amyloidosis (Fig.3). 

On polarizing microscope, these deposits exhibited ‘apple-green’ birefringence. In our case, X-ray chest, X-ray spine, X-ray skull, ECG, rectal biopsy, bone marrow biopsy and abdominal fat pad aspiration cytology were done to rule out systemic amyloidosis, which were normal. 

Our patient presently responded well to transurethral resection without recurrence since last one year and is advised cystoscopy annually.

## Discussion

Historically, ‘amyloid’ term was coined by Virchow in 1854 meaning ‘starch-like’ to describe tissue deposits that stained with iodine solutions. Amyloidosis has three major forms based on which it is classified into primary (AL), secondary (AA) and hereditary (ATTR). Plasma cell dyscrasias lead to primary amyloidosis and secondary amyloidosis is commonly associated with chronic inflammation and systemic diseases like Rheumatoid arthritis, Crohn’s disease (8, 9). 

Primary localized VA is a very rare entity, which was first coined by Solonis (1897) at autopsy (3). Though exact etiology is unknown, immunological factors are considered to play a role for bladder amyloidosis. (4)

VA is generally seen to occur in generalized or localized tumefactive form (4). The latter has been reported in diverse sites particularly, the respiratory tract, skin, mucus membranes and the heart. In the genito-urinary tract, primary localized involvement of renal pelvis, ureter, urinary bladder (most common), prostate, seminal vesicles, urethra, penis and testes may occur (4). 

Primary localized VA is seen to occur in advanced ages with mean age of 55 years and equal male: female ratio (8). Rectal biopsy, bone marrow biopsy and abdominal fat pad aspiration done to rule out systemic amyloidosis, are negative in primary localized VA (5, 9) like in our case. Electron microscopy shows non-branching thin amyloid fibrils, which was not done in our case (6). New technique of serum amyloid P component scintigraphy can show uptake in organs and helps rule out systemic amyloidosis (7), which was not done in our case.

Present case of primary localized VA was misdiagnosed as bladder tumor clinically, on cystoscopy and imaging techniques. Experience is shared by others (1-9). Gross hematuria was seen in nearly 60% cases, 20% with storage urinary tract symptoms like dysuria/increased micturition, 20% with both (1). Our case had only hematuria (1, 5). Our patient did not have dysuria unlike 2/5 cases reported by Gupta P et al (5). 

Such youngest reported primary VA case was a 25-year-old female with associated endometriosis, while our case was a 40-year-old man. Our case did not have any associated malignancy like squamous cell carcinoma, basal cell carcinoma (1) or associated hypertension, increased micturition due to diabetes mellitus (7), or prostatomegaly (7, 9) or hydro-ureteronephrosis with transitional cell carcinoma (3) and renal cysts (1).

CT-scan and MRI are generally not diagnostic of VA (1), whereas cystoscopic appearances have been variably described as fleshy, nodular, protuberant or polypoidal lesions which generally are interpreted as invasive bladder carcinoma with associated urethral involvement (1). 

Our solitary bladder lesion was located in right lateral wall of bladder. Similar affection of side was noted in 13 out of 31 cases of primary localized amyloidosis reviewed by Malek et al (10) and other studies (1, 3, 4, 9). The most recent studies, favored the site of posterior bladder wall-68% (3, 4, 8), followed by the trigone-26% (7, 8). “Multiple amyloid deposits are more common (65%) as compared to single area of involvement in 26%, and diffuse involvement of bladder in 10% of cases” (5,8). Our case had a single lesion (1,5) even though more than two/ multiple lesions have been reported mostly (3, 5, 7).

On cystoscopy, rarely anterior wall of urinary bladder (7) have been the site of localized VA. Erythematous lesion was seen in our study like in case reported by DeSouza MA et al (7). A single case (8) had posterior wall involvement with urethral amyloid deposit too, not seen in our case. 

Cystoscopic differential diagnoses for primary vesical amyloidosis are malignancy, schistosomiasis, hemorrhagic and interstitial cystitis, thus making biopsy as ‘gold standard’ in diagnosis. 

Exact diagnosis of VA can be made on biopsy, supplemented with special stains as radiological examination are seldom diagnostic (7). We received a biopsy from the lesion that showed just mucosa and submucosa without the muscle layer, the latter is affected in primary localized vesical amyloidosis (7). We had foreign-body giant cells with chronic mononuclear infiltrate along with amyloid deposits, so differential diagnosis of fungal/schistosomiasis parasitic infestation (7) was also considered. However, this diagnosis was ruled out by absence of such definite pathogens in biopsy.

 Gaitonde et al. have described presence of amyloid deposits in superficial as well as deep blood vessels (2). Systemic amyloidosis shows heavy deposits in vascular sites while primary localized VA spares the blood vessels (10). In present case, blood vessels were not affected by amyloidosis. The deposit was predominantly interstitial and with giant cell and lymphocytic reaction to it. On contrary to above statements, blood vessels are rarely affected like in the two reported cases of primary localized VA (9). 

In view of absence of any pre-disposing chronic illness, localized nature of amyloid deposit, Congo-red staining and its resistance to KMnO_4_ pre-treatment, generally the diagnosis of primary amyloid of urinary bladder is offered (5). The patient is treated with TUR and histopathologic evaluation to exclude malignancy. In most cases, this ‘gold standard’ therapy is adequate to control the lesion like in our case (8). 

Isolated reports of medical regimens like oral colchicines, cepharanthin, nitrofurazone and intra-vesical dimethyl-sulphoxide installation, intra-vesical KMnO_4_ as primary and adjuvant therapy show limited success rates (7, 9). 

VA cases are advised careful follow-up as recurrence may occur within few months to several years in 50% of cases. To our knowledge, there have been only three reported cases of bladder malignancy in patients with primary vesical amyloidosis monitored over a long period (1). 

## Conclusion

Primary localized VA should be considered as a differential diagnosis of bladder carcinomas, especially in elderly cases of long-standing underlying illness with recurrent hematuria. CT-scan and MRI are generally not diagnostic. Correct diagnosis relies on clinical alertness and use of special staining during ‘gold standard’ histopathological examination. For differential diagnosis of primary and secondary amyloidosis, KMnO_4_ pre-treatment before Congo-red staining can be used. In diagnosed and treated cases of primary localized VA, regular life-long follow-up with regular cystoscopic evaluation is must to rule out subsequent recurrence and concomitant tumors. 

## Conflict of Interests

The authors declare that there is no Conflict of Interests. 
